# The Impact of Grit on Nurses' Job Performance: Evaluating Chained Mediation through Perceived Social Support and Self-Esteem

**DOI:** 10.1155/2024/6388800

**Published:** 2024-02-27

**Authors:** Xiqin Liu, Jingguang Li, Song Wang

**Affiliations:** ^1^Department of Radiology and Huaxi MR Research Center (HMRRC), West China Hospital, Sichuan University/West China School of Nursing, Sichuan University, Chengdu, China; ^2^College of Teacher Education, Dali University, Dali, China

## Abstract

**Background:**

Nurses play a critical role in the medical workforce during the COVID-19 pandemic while facing various difficulties and challenges. Grit, social support, and self-esteem are important psychosocial factors influencing job performance. However, few studies have explored the relationships among these factors in nurses.

**Aim:**

This study aimed to examine the association between grit and nurses' job performance during the COVID-19 pandemic and to explore the potential chain mediation through perceived social support and self-esteem.

**Methods:**

A cross-sectional survey design was employed. From January 2021 to May 2022, a total of 709 Chinese nurses in Chengdu and Kunming completed a web-based cross-sectional survey, which included standard assessments on grit, perceived social support, self-esteem, and job performance as well as Big Five personalities. The chain mediation model was tested using the PROCESS macro program in the SPSS software.

**Results:**

There was a moderate-to-large correlation (*r* = 0.40, *p* < 0.001) between grit and job performance in Chinese nurses. Furthermore, grit was indirectly linked to job performance through the chain mediating effect of perceived social support and self-esteem (all *p* < 0.05). These findings persisted even when Big Five personalities were included as additional controlling variables.

**Conclusions:**

This study reveals a stable link between grit and job performance among Chinese nurses and highlights the potential role of perceived social support and self-esteem in mediating this link. *Implications for Nursing Management*. Nursing managers can improve nurses' grit level and provide a supportive organizational environment conducive to enhancing self-esteem and thereby promoting their job performance.

## 1. Introduction

Accounting for the largest portion of the medical workforce [1], nurses play an increasingly important role in the healthcare system, especially highlighted by the COVID-19 pandemic [2]. The public labeled nurses as “heroes” because of their courage, bravery, commitment, resilience, and persistence during the pandemic [3]. Their work not only relates to the lives and health of patients but also plays a vital role in the management of public health and healthcare resources for society as a whole. Nevertheless, nurses had to face various difficulties and challenges such as working in high-risk environments, heavy workloads, insufficient understanding and support from managers, and inadequate specialized training, which could result in burnout, lack of personal accomplishment, and lower efficiency and performance at work [4].

The complex situations during the pandemic exacerbated this dilemma and brought about organizational crises [5]. For instance, a survey by the American Nurses Foundation revealed that nearly 90% of the nurses reported staff shortages in their organizations and more than half of the respondents reported negative feelings such as being extremely stressed and undervalued during the COVID-19 pandemic [6], which may further lead to decreased personal performance at work [7]. In this context, probing the psychosocial factors that may help nurses work effectively and improve performance addresses an important issue in nursing management and practice.

The active field of positive psychology offers novel insights into improving job performance [8], and one promising positive psychological construct is grit, a personality quality that can help explain why two individuals with the same level of intellectual ability are often observed to perform differently in a given field [9, 10]. Previous studies on different groups have indicated that grit positively impacts personal performance [10–13]. However, whether grit affects job performance in nurses and its underlying psychological mechanism remains unclear. Therefore, this study focused on the association between grit and job performance in nurses during the COVID-19 pandemic and explored the potential mediation mechanism underlying this association.

### 1.1. Grit and Job Performance

Grit, defined as perseverance and passion for long-term goals, is a nonintellectual trait essential to personal success and performance in various domains [10]. Gritty individuals are typified by working hard towards challenges with sustained efforts and interests despite failures, adversity, and plateaus [9, 10]. The well-established conservation of resources (COR) theory suggests that grit is an important personal resource that can facilitate goal achievement and promote personal growth and development [14, 15]. It is thought that individuals with high levels of grit are able to make better use of their abilities because they are less distracted by short-term goals and less discouraged by failures and setbacks that are commonly encountered in many performance areas [16], which can lead to better performance in those areas. Numerous empirical studies have demonstrated the positive effect of grit on personal performance in a wide range of fields covering students' academic performance [13, 17], employees' job performance [11, 18, 19], performance in military [20], and sports [21]. In particular, grit has been found to predict both the academic and clinical performance of nursing students after controlling for potential confounders including the year of study and demographic factors [13]. Despite the well-documented relationship between grit and performance in these areas, the impact of grit on job performance in nurses has not been robustly determined. Moreover, grit has been proposed as an essential trait of nurses during a disaster to enable them to complete the arduous tasks required [22]. Hence, the first aim of this study was thus to determine the association between grit and job performance in a sample of Chinese nurses during the COVID-19 pandemic.

### 1.2. Grit, Perceived Social Support, Self-Esteem, and Job Performance

Understanding how grit affects job performance is important for nursing managers developing effective strategies to improve nurses' job performance. Some have argued that goal pursuit and attainment are outcomes of interaction between personal resources and the environment [23, 24]. A qualitative study found that the social support system (e.g., family, friends, significant others, superiors, or colleagues) is a major contributor of grit in achieving personal and work goals in Asian graduate students [25]. When people feel supported by others in their pursuit of long-term goals, they may display greater levels of perseverance, resulting in improved performance [25]. In return, gritty individuals are more likely to attract social support due to their unwavering dedication and passion, which may exert a positive effect on performance [24]. It has been demonstrated that perceived social support is more predictive and functional than received social support [26], even though they are highly correlated [27]. Perceived social support is defined as the perception of the available support from one's social networks [26]. Previous studies have found positive correlations between grit and perceived social support in adolescents [28] and nurses [29]. Moreover, there are also positive associations between perceived social support and nurses' task performance [30], particularly during the COVID-19 pandemic [31]. According to the Job Demands-Resources (JD-R) model [32], social support is a form of job resources provided to employees to deal with the job demands which can help them stay healthy and is therefore closely related to job performance [33]. Low levels of perceived social support have been reported to cause emotional exhaustion in working women during COVID-19 which may relate to the job performance [34]. On the other hand, it is suggested that certain personalities, e.g., grit, can help employees cope with job demands and increase their job resources, which in turn can lead to better job performance [35]. Hence, in investigating the relationship between grit and job performance, perceived social support can be regarded as an important mediator at the external influence level [25].

Self-esteem is another personal resource that helps motivate and facilitate goal attainment in COR theory, which has been shown to be an important indicator of personal performance [36–38]. Self-esteem refers to an individual's overall evaluation of their own worth, competence, and value [39]. High self-esteem stimulates self-potential [40] and is associated with many positive behaviors and outcomes such as enhanced learning motivations [41], less antisocial behavior [42], higher life satisfaction [43], and better physical and mental health [42, 44]. Importantly, self-esteem is one of four dispositional traits (i.e., self-esteem, generalized self-efficacy, locus of control, and emotional stability) that are significantly related to job satisfaction and job performance according to Judge, Locke, and colleagues' theory of core self-evaluations [45] and exhibits the highest correlation with job performance among the four traits [37]. Korman [46] self-consistency theory suggests that individuals with high self-esteem will perform effectively in order to maintain a positive self-image. On the other hand, prior research suggests that self-esteem shows a medium-sized association with grit [40, 47], and this may be related to the possibility that during the process of pursuing long-term goals with passion and persistence, gritty individuals can gain a better understanding about themselves and be more satisfied with themselves [48, 49], which may contribute to higher levels of self-esteem and better job performance. Thus, self-esteem may be an internal factor that mediates the relationship between grit and job performance.

With respect to the relationship between perceived social support and self-esteem, it has been argued that social support is a critical component for maintaining individuals' self-esteem [31], while self-esteem can reflect the quality of interpersonal relationships based on the sociometer hypothesis [50, 51]. Several studies have found a positive correlation between perceived social support and individuals' self-esteem [43, 52, 53]. A recent study shows that the failure to receive online social support during COVID-19 can result in reduced self-esteem and increased loneliness [54]. According to the sociometer theory, when one's relational deficiencies are detected, self-esteem will be negatively affected in an automatic, unconscious way as an “internal monitor” [55]. By contrast, high perceived social support from family, friends, and significant others can enhance university students' self-esteem, promote their academic achievements, and mitigate their emotional exhaustion [47]. Furthermore, self-esteem is found to mediate the relationship between perceived social support and academic achievement [48, 56] as well as the intention to stay in nurses [57]. Previous studies have indicated that perceived social support and self-esteem sequentially mediate the association between Internet use and loneliness [54, 58]. Based on the above findings and theories, it is plausible to explore the chain mediation of perceived social support and self-esteem in the relationship between grit and nurses' job performance. The second aim of this study was thus to examine the indirect effect of grit on job performance with perceived social support and self-esteem as the intervening variables.

### 1.3. The Current Study

This study aimed to reveal the relationship between grit and nurses' job performance and particularly examine the sequential mediating roles of perceived social support and self-esteem in this relationship. The present study extends previous research in several respects. First, prior investigations into the link between grit and performance focused mainly on the academic and other professional domains [11, 17, 21], whereas few studies have tested it in nurses [59, 60]. Given the large number of Chinese nurses (i.e., 4.7 million as of 2020) and their heavy workloads and unprecedented pressure in the COVID-19 pandemic [61], it is essential to explore how Chinese nurses' grit influences their job performance. Second, although previous studies and theories suggest that social support and self-esteem may play a mediating role in the link between grit and job performance, no research has directly examined the mediating effect, especially the sequential mediating effect, of perceived social support and self-esteem between grit and job performance. Third, previous research generally did not control for similar psychological constructs such as Big Five conscientiousness when examining the relationship between grit and performance, which may be a reason for the inconsistent results of previous studies [10, 16]. Therefore, this study used well-validated measurements to investigate the intercorrelations among grit, job performance, perceived social support, self-esteem, and Big Five personality traits in a large population of Chinese nurses (*n* = 756) during the COVID-19 pandemic. Then, we constructed a serial mediation model to test the chain mediating effects of perceived social support and self-esteem in the grit-job performance association and moreover tested the specificity of the results by controlling for effects of Big Five personalities in this model given that Big Five traits have been shown to relate robustly to grit [48] and job performance [62]. These analyses can help elucidate the mechanism and specificity of the impact of grit on nurses' job performance. Based on previous research, we proposed the following hypotheses:

**Hypothesis 1 .**

Grit is significantly and positively related to job performance in nurses

**Hypothesis 2 .**

Perceived social support mediates the relationship between grit and nurses' job performance

**Hypothesis 3 .**

Self-esteem mediates the relationship between grit and nurses' job performance

**Hypothesis 4 .**

Perceived social support and self-esteem sequentially mediate the relationship between grit and nurses' job performance

## 2. Methods and Materials

The present study was implemented using a cross-sectional survey design. We adhered to Strengthening the Reporting of Observational Studies in Epidemiology (STROBE) guidelines and methodology in reports of cross-sectional studies (see Supplementary Information: Table S1).

### 2.1. Participants and Procedures

A total of 756 nurses from several hospitals in Chengdu and Kunming in Southwest China through convenience sampling participated in this study between January 2021 and May 2022. Forty-seven participants who failed to pass the bogus items (e.g., I have five fingers on my left hand) that had only one correct answer in the survey [63] were excluded from analyses, resulting in a final sample of 709 respondents (643 females, age = 18–55 years, mean age = 31.74 ± 7.38 years) with an effective response rate of 93.78% [64]. The current sample size was far greater than the estimated sample size (*n* = 207) to detect medium-sized effects based on standard power analysis while considering 20% of incomplete surveys [65, 66]. The inclusion criteria were (a) obtaining a professional qualification certificate for nurses in the People's Republic of China, (b) having at least 1 year of experience in clinical nursing or nursing management, (c) having no previous or current diagnosis of mental illness or drug or alcohol dependence, and (d) having the skills to complete the questionnaires online. Trainee nurses, standardized training nurses, and nurses who were unable to complete the survey were excluded. Notably, none of the participants were infected with COVID-19 proved by their nucleic acid testing report.

The questionnaires were distributed through online recruitment to designated investigators in each hospital who had been trained by the researchers, in compliance with quarantine guidelines to mitigate the spread of COVID-19 infection. After signing an online informed consent to their voluntary participation in the study, each participant completed the questionnaires independently under the guidance of the investigator. The investigators needed to answer questions for participants based on uniform guidelines. The survey was anonymous, and data confidentiality was assured. All measures were written in simplified Chinese. This study was approved by the local Ethics Committee of West China Hospital of Sichuan University.

### 2.2. Measures

#### 2.2.1. Short Grit Scale

The scale is a self-report instrument originally developed and validated by Duckworth and Quinn [11] to measure grit. It includes two subscales: consistency of interest (e.g., “New ideas and projects sometimes distract me from previous ones”) and perseverance of effort (e.g., “I finish whatever I begin”). Each subscale consists of four items, and participants were required to rate their agreement to each item on a five-point Likert scale ranging from 1 (not like me at all) to 5 (very much like me). The Chinese version of this scale was translated and validated by Li and colleagues (2018) using a back-translation process, which proved to have good reliability and validity among different populations [67–69]. All items in the consistency of interest subscale are negatively phrased, and the item responses are reverse-coded for scoring. A total score was obtained by summing the subscale scores, with higher total scores indicating a higher level of grit. Given that previous studies have demonstrated that the total score was a better predictor of personal success in the most demanding domains than either factor alone [11, 70], we focused on the total score in the current analyses. Cronbach's *α* of this scale in this study was 0.75, indicating adequate internal reliability.

#### 2.2.2. Task Performance Scale

We used the five-item Task Performance Scale for assessing in-role job performance [71, 72]. Participants were asked to indicate the extent to which they agreed or disagreed on a seven-point Likert scale (1 = “strongly disagree” to 7 = “strongly agree”) with the statements such as “I can always complete the duties specified in my job description.” Task performance is represented by summing the scores; the higher the score, the better the task performance. The scale showed acceptable psychometric properties in Chinese samples [67, 73]. In this study, Cronbach's *α* was 0.96, indicating satisfactory internal reliability.

#### 2.2.3. Multi-Dimensional Scale of Perceived Social Support

The scale was used to measure an individual's perception of social support from various sources, i.e., significant others, family, and friends [74]. The scale consists of 12 items, e.g., “My family really tries to help me.” Participants rate their agreement with each statement using a seven-point Likert scale ranging from 1 (very strongly disagree) to 7 (very strongly agree). The scores from the items are summed to provide a total score for each dimension and an overall score for perceived social support. The scale has shown good reliability and validity in Chinese populations [58, 75]. As in previous studies [48, 76], the present study only focused on the overall score in the analyses. In the current study, Cronbach'*α* of this scale was 0.94, indicating satisfactory internal reliability.

#### 2.2.4. Rosenberg Self-esteem Scale

The scale was used to assess self-esteem [39], which consists of 10 items measuring an individual's overall evaluation of himself/herself. Participants are asked to indicate their level of agreement or disagreement with each statement using a four-point Likert scale ranging from 1 (strongly disagree) to 4 (strongly agree). It includes items such as “I am able to do things as well as most other people.” Total scores can range from 10 (lowest self-esteem) to 40 (highest self-esteem). The scale has good reliability and validity in Chinese populations [56, 77]. In this study, Cronbach's *α* was 0.88, indicating adequate internal reliability.

#### 2.2.5. Big Five Inventory

To exclude the potential effects of general personalities on the associations among grit, perceived social support, self-esteem, and job performance, we used a 44-item Big Five Inventory to evaluate the personality traits including openness, agreeableness, conscientiousness, extraversion, and neuroticism [78]. Each item is assessed on a five-point Likert scale ranging from 1 (strongly disagree) to 5 (strongly agree). The scale has been translated and validated across different countries and cultures [79, 80]. The Chinese version has demonstrated adequate reliability and validity [79, 81]. In this study, Cronbach's *α*s of the five dimensions were 0.78 (openness), 0.72 (agreeableness), 0.80 (conscientiousness), 0.72 (extraversion), and 0.78 (neuroticism), indicating adequate internal reliability.

### 2.3. Data Analysis

All behavioral data were analyzed using the statistical software SPSS 25.0 (SPSS Inc., Chicago, IL, USA). There are no missing data due to the settings of the web-based survey. First, to examine the effect of common method deviation that may arise from the measurement method regarding self-reported scales and can lead to inaccurate results [82, 83], Harman's single-factor tests were conducted on all items of the four scales [84]. The participants' sociodemographic characteristics and major variables were analyzed as descriptive statistics (i.e., mean and standard deviation (SD)). Pearson correlation analyses were performed to investigate the bivariate correlations among the measurements.

To test the mediating effects of perceived social support and self-esteem in the relationship between nurses' grit and job performance, we used a bias-corrected bootstrapping method with PROCESS macro implemented in SPSS [85]. Mode 6 was chosen for the serial mediation analysis. We tested our hypothesized chain mediation model in which grit (*X*) increases perceived social support (M1), which affects self-esteem (M2), which in turn leads to higher job performance (*Y*), while controlling for sex, age, education, professional title, marital status, and working years (as well as Big-Five factors). Bias-corrected confidence intervals for the indirect effect were calculated using 5,000 bootstrapped samples. This analysis also tests each mediator separately to assess the contribution of the simple mediations to the indirect effect on the relationship between *X* and *Y*. An empirical 95% confidence interval that did not contain 0 signified that the mediating effect was statistically significant [86].

## 3. Results

### 3.1. Common Method Bias

The results indicated that there were seven eigenvalues whose values exceeded 1 and the variance explained by the first eigenvalue was 33.37% (<40%), indicating that this study did not suffer from serious methodology bias [84].

### 3.2. Descriptive Statistics and Correlation Analysis

Table 1 presents the frequency/percentage or mean, SD, and range for sociodemographic characteristics. Most of the participants were females (643 females, 90.7%), and the average age for all participants was 31.74 ± 7.38 years old. 398 nurses (56.1%) had a bachelor's degree; 483 nurses (68.1%) were married; 366 nurses (51.6%) had a primary title; and the average total work experience was 10.67 ± 8.12 years.

Descriptive statistics and bivariate correlations for all measurements are shown in Table 2. As expected, higher grit was positively associated with higher perceived social support, self-esteem, and job performance (*ps* < 0.001). Perceived social support, self-esteem, and job performance were also intercorrelated (*ps* < 0.001). Moreover, grit was associated with all Big Five personality traits (*ps* < 0.001).

### 3.3. Chain Mediation Model Analysis

A serial mediation analysis was conducted to test the effect of perceived social support and self-esteem as multiple sequential mediators in the indirect relationship between grit and job performance in nurses via Model 6 in SPSS macro. Grit and job performance were entered as the independent (*X*) and dependent (*Y*) variables, respectively; perceived social support (*M*1) and self-esteem (*M*2) were added as mediators; sex, age, education, professional title, marital status, and working years were included as covariates.

**Figure 1:**
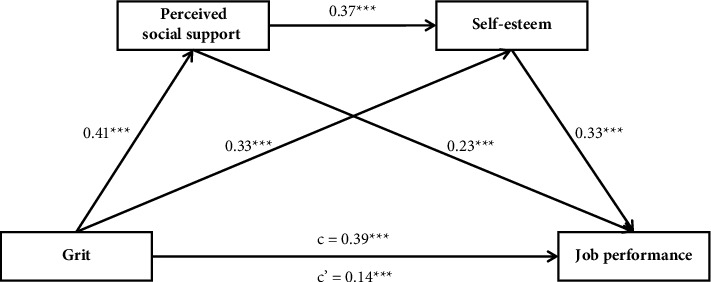
Model of the mediation role of perceived social support and self-esteem in the relationship between grit and job performance. Standardized regression coefficients were displayed in the path diagram; c, total effect; c', direct effect; ^*∗∗∗*^*p* < 0.001, ^*∗∗*^*p* < 0.01. Sex, age, education, professional title, marital status, and working years were treated as covariates in the model.

As shown in Table 3 and Figure 1, the total effect of grit on job performance (*β* = 0.39, *p* < 0.001) decreased when the mediators were included in the model (*β* = 0.14, *p* < 0.001), suggesting that the effect of grit on job performance was partially mediated by perceived social support and self-esteem. Bootstrap estimation procedure (*n* = 5000) indicated that the indirect effects for this model were statistically significant (indirect effects = 0.55, *SE* = 0.03, 95% CI = [0.21, 0.31], accounting for 64.1% of the total variance; Table 3). The indirect effects were generated through three paths: Path 1 consisting of grit⟶perceived social support⟶job performance (indirect effect = 0.09, 95% CI = [0.05, 0.14]), Path 2 consisting of grit⟶self-esteem⟶job performance (indirect effect = 0.11, 95% CI = [0.07, 0.15]), and Path 3 consisting of grit⟶perceived social support⟶self-esteem⟶job performance (indirect effect = 0.05, 95% CI = [0.03, 0.07]), which were all statistically significant. Hence, there is a chain mediation of social support and self-esteem as well as separate mediating effects of each on the relationship between grit and job performance in nurses.

### 3.4. Specificity of Findings

To test the specificity of the findings, we furthermore added Big Five personality traits, i.e., openness, agreeableness, conscientiousness, extraversion, and neuroticism, as additional controlling variables in the serial mediation model. The analysis yielded robust results (see Supplementary Information: Table S2 and Figure S1).

## 4. Discussion

In the present study, we investigated the relationship between grit and job performance in a sample of Chinese nurses during the COVID-19 pandemic, as well as the mediating role that perceived social support and self-esteem played in this relationship. The results revealed that grit not only directly influenced job performance but indirectly affected job performance through perceived social support and self-esteem separately and sequentially, and these findings were independent of Big Five personalities, all of which may provide an insightful understanding of underlying mechanisms that explain the influence of grit on job performance.

The positive relationship between grit and job performance found in the current sample is consistent with the findings of previous studies on this relationship [18, 19, 60]. Particularly, the present study verified this association in a large population of Chinese nurses during the pandemic even after controlling for the effects of Big Five personality traits, which extends the generalizability, specificity, and ecological validity of previous findings on this link. This finding agrees with the well-established COR theory, which proposes that grit is one of the personal resources linked to work goal progress [14, 24]. Prior research has shown that grit is associated with less burnout in healthcare professionals and students [87, 88], which facilitates long-term work and stability of nursing staff [88]. The increased job demands (e.g., surges in patient volume) during the pandemic led to healthcare professionals' burnout and exhaustion [2], which is found to impact objective and subjective work performance in nurses [89]. High levels of grit may protect nurses from burnout and persevere through challenges [90] and therefore affect nursing job performance. Notably, the grit-job performance association was independent of Big Five personality traits, which supports the specificity of the current findings. Big Five traits have long been found to predict success and achievements [11, 78, 91]. In particular, Big Five conscientiousness is strongly correlated with grit and is a robust predictor of job performance [11, 62], yet Grit-S predicts achievement outcomes over and beyond conscientiousness and other Big Five traits [10, 11, 48, 92], which aligns with the present finding. Our study provided further evidence that grit captures a unique aspect of perseverance and passion that is distinct from the traditional personality dimensions, which appears to specifically predict job performance in nursing contexts. Nevertheless, based on the too-much-of-a-good-thing (TMGT) framework in management proposed by Pierce and Aguinis [93], the beneficial role of psychological resources (e.g., grit) may be reversed into a negative factor of performance when grit is at a very high level; thus, future studies are needed to test the curvilinear relationship between grit and nurses' job performance [24].

The finding that perceived social support and self-esteem separately mediate the relationship between grit and job performance in nurses supports the JD-R model and COR theory. According to the JD-R model, social support is a type of job resource and grit and self-esteem can be deemed as personal resources that ultimately improve the job performance of working people [33], and external supports supplement internal motivation to increase their ability to perform well in their jobs. Hobfoll's [14] COR theory posits that people tend to acquire, maintain, and avoid losses of valuable resources, including objects (e.g., food), environmental conditions (e.g., interpersonal relationships), personal characteristics (e.g., grit and self-esteem), and energies (e.g., time) that serve as means for goal attainment. The COR theory further integrates the impact of perceived social support as a moderator of the relationship between grit and goal progress [24]. Research shows that gritty nurses can improve their meaning in life through perceived social support [29]. It may be that social support provides emotional assistance to help gritty individuals cope with workplace stress and adversity as they continue to pursue long-term goals, which is conducive to maintaining their performance levels. In particular, gritty nurses tend to feel a high level of social support because they use more positive coping strategies to deal with stress such as seeking and appreciating social support [94], which may enhance their perceived social support. Consequently, nurses with a high level of perceived social support are less likely to experience burnout and more likely to have increased overall well-being, which can lead to improved job performance as they are better able to engage in their work even during the pandemic [30, 95]. On the other hand, self-esteem, as a core self-evaluation trait, has been demonstrated to show the highest correlation with job performance as compared to other core traits [37] and is also positively related to grit [40, 43, 96]. Prior research has found self-esteem as a mediator in the link between grit and employees' life satisfaction [47]. The current study extends previous studies by revealing an indirect effect of grit on nurses' job performance through self-esteem. The mediating role of self-esteem in the grit-job performance relationship may be because gritty nurses may receive more positive feedback and reinforcement due to their persistence and passion, which can further enhance their self-esteem and encourage them to continue to achieve high job performance [48, 49].

The most important contribution of this study is elucidating the chain mediating effect of perceived social support and self-esteem on the relationship between grit and nurses' job performance. High levels of grit are associated with the perception of greater perceived social support, which in turn fosters higher self-esteem and ultimately improves nurse performance, highlighting the potential usefulness of social support as an external source and the resulting self-esteem as a personal resource to improve job performance in gritty people. According to the sociometer theory [55], self-esteem is an internal measure to monitor one's interpersonal relationships and success. Feeling supported by others can contribute to individuals' overall sense of self-worth, value, and confidence in their abilities, whereas a lack of support from social relations makes individuals feel devalued and rejected [97], which can lead to negative self-evaluations and low self-esteem. Studies found that perceived organizational support can significantly increase nurses' self-esteem [53, 98]. Social support has long been recognized as an important coping strategy during times of crisis, and reduced social support is associated with nurses' burnout [4] and traumatic stress [99] during the COVID-19 pandemic. Thus, during a time full of stress and uncertainty, gritty nurses might actively seek and utilize their social support networks to maintain a high perception of social support, which can boost their self-esteem and in turn lead to better job performance during the pandemic.

## 5. Limitations of the Study

This study has some limitations. First, the study used a cross-sectional design which did not permit inference about the causality relations; thus, caution is needed to interpret the current finding, and the feasibility of the findings for use in nursing management warrants further investigation. Future studies may also test the mediating models using a longitudinal design tracking the changes of these variables over time to draw robust conclusions about causality [100]. Second, the data were collected only through self-report instruments. Although these questionnaires were selected for their good reliability and validity, self-report measures are vulnerable to biases, such as social desirability; future studies should incorporate more objective evaluations [101] and experimental tasks to precisely measure these variables. Third, the present study only measured perceived social support from family, friends, and significant others and did not assess perceived organizational support [98], which may play a particularly important role in the intensive work during the pandemic [102]. Fourth, this study only focused on Chinese nurses sampled from several local hospitals in Southwest China, and future studies need to expand the survey area (e.g., other regions of China or other countries) to improve the generalizability of the findings. Finally, previous neuroimaging studies have identified the neural substrates of grit [92, 103] and determined the neuroanatomical basis linking perceived social support to self-esteem [104], yet how these brain markers predict job performance remains uninvestigated; future studies are invited to explore the neural mechanisms underlying the associations among these variables in the framework of organizational cognitive neuroscience [105, 106].

## 6. Implications for Nursing Management

Our findings have critical implications for nursing management. The chain mediation provides new insights into enhancing grit to improve job performance. Specifically, at the organizational level, nursing managers should implement personal education about the importance of grit in nurses, which can help them cope with stress and challenges when facing obstacles and difficulties at work. Second, it is necessary to provide unconditional organizational support to nurses and build support teams to expand their knowledge and skills as well as share their experiences and emotions, which may be an essential job resource that helps form positive feelings and evaluations of themselves [54] so as to increase their job performance. Moreover, psychological training programs aiming at fostering grit and self-esteem are suggested to be developed and adopted, which may also be conducive to nurses' job performance, especially during a crisis like the COVID-19 pandemic.

## 7. Conclusions

The current study investigated the association between grit and nurses' job performance during the COVID-19 pandemic and explored the chain mediation through perceived social support and self-esteem. The results showed that grit not only directly affects job performance but also has indirect effects through perceived social support and self-esteem, both independently and sequentially. These findings were not affected by Big Five personalities as well as sociodemographic characteristics, showing a specific nature to some degree. Our study has important implication for nursing management to improve nurses' job performance.

## Figures and Tables

**Table 1:** Sociodemographic characteristics of the participants.

Variable	Mean ± SD (range) *N*%
Sex	
Female	643 (90.7%)
Male	66 (9.3%)
Age (years)	31.74 ± 7.38 (18–55)
Educational level	
Graduate degree	4 (0.6%)
Bachelor degree	398 (56.1%)
College degree	270 (38.1%)
Secondary vocational degree	37 (5.2%)
Marital status	
Married	483 (68.1%)
Unmarried	198 (27.9%)
Divorced	26 (3.7%)
Widowed	2 (0.3%)
Professional title	
Vice senior	10 (1.4%)
Intermediate	170 (24.0%)
Primary	366 (51.6%)
None	163 (23.0%)
Length of nursing work (years)	10.67 ± 8.12 (1–39)

*Note. N*, number; SD, standard deviation.

**Table 2:** Descriptive statistics and bivariate correlations of study measures.

Measure	Mean ± SD	Range	1	2	3	4	5	6	7	8
1. Grit	27.03 ± 4.14	17–40	—							
2. Perceived social support	66.23 ± 10.69	21–84	0.41	—						
3. Self-esteem	31.03 ± 4.30	16–40	0.50	0.53	—					
4. Job performance	29.20 ± 4.21	5–35	0.40	0.46	0.52	—				
5. Extraversion	25.16 ± 4.19	11–37	0.31	0.31	0.37	0.17	—			
6. Agreeableness	35.34 ± 3.94	22–45	0.43	0.46	0.46	0.36	0.21	—		
7. Conscientiousness	32.92 ± 4.60	14–45	0.56	0.28	0.50	0.39	0.30	0.49	—	
8. Neuroticism	22.94 ± 4.83	9–39	−0.45	−0.36	−0.45	−0.25	−0.45	−0.46	−0.49	—
9. Openness	32.10 ± 4.86	19–48	0.39	0.33	0.35	0.27	0.42	0.28	0.35	−0.36

*Note.* All correlation coefficients were statistically significant at the *p* < 0.001 level. SD, standard deviation.

**Table 3:** Total, direct, and indirect effects of the mediation model.

Effect	Effect size	Bootstrap SE	Bootstrap 95% CI
Total effect (grit ⟶ job performance)	0.39	0.03	[0.33, 0.47]
Direct effect (grit ⟶ job performance)	0.14	0.04	[0.07, 0.21]
Indirect effect	0.25	0.03	[0.21, 0.31]
Grit ⟶ perceived social support ⟶ job performance	0.09	0.02	[0.05, 0.14]
Grit ⟶ self-esteem ⟶ job performance	0.11	0.02	[0.07, 0.15]
Grit ⟶ perceived social support ⟶ self-esteem ⟶ job performance	0.05	0.01	[0.03, 0.07]

*Note.* SE, standard error; CI, confidence interval. Sex, age, education, professional title, marital status, and working years were treated as covariates in the model.

## Data Availability

West China Hospital of Sichuan University has an institutional commitment to data sharing. To get access to the data and comply with the terms of our research ethics committee approval, an application to the corresponding author will be required, specifying the geographical extent of sharing.

## Supplementary Materials

Supplementary Materials Supplementary 1. Table S1. STROBE Statement: Checklist of items that should be included in reports of cross-sectional studies. Supplementary 2. Table S2. Total, direct, and indirect effects of the mediation model. Supplementary 3. Figure S1. Model of the mediation role of perceived social support and self-esteem in the relationship between grit and job performance.
